# Short term culture of breast cancer tissues to study the activity of the anticancer drug taxol in an intact tumor environment

**DOI:** 10.1186/1471-2407-6-86

**Published:** 2006-04-07

**Authors:** Heiko van der Kuip, Thomas E Mürdter, Maike Sonnenberg, Monika McClellan, Susanne Gutzeit, Andreas Gerteis, Wolfgang Simon, Peter Fritz, Walter E Aulitzky

**Affiliations:** 1 Dr. Margarete Fischer-Bosch Institute of Clinical Pharmacology, Stuttgart, Germany; 2 Department of Gynecology, Robert Bosch Hospital, Stuttgart, Germany; 3 Department of Diagnostic Medicine, Pathology, Robert Bosch Hospital, Stuttgart, Germany; 4 2^nd ^Department of Internal Medicine, Oncology and Hematology, Robert Bosch Hospital, Stuttgart, Germany

## Abstract

**Background:**

Sensitivity of breast tumors to anticancer drugs depends upon dynamic interactions between epithelial tumor cells and their microenvironment including stromal cells and extracellular matrix. To study drug-sensitivity within different compartments of an individual tumor *ex vivo*, culture models directly established from fresh tumor tissues are absolutely essential.

**Methods:**

We prepared 0.2 mm thick tissue slices from freshly excised tumor samples and cultivated them individually in the presence or absence of taxol for 4 days. To visualize viability, cell death, and expression of surface molecules in different compartments of non-fixed primary breast cancer tissues we established a method based on confocal imaging using mitochondria- and DNA-selective dyes and fluorescent-conjugated antibodies. Proliferation and apoptosis was assessed by immunohistochemistry in sections from paraffin-embedded slices. Overall viability was also analyzed in homogenized tissue slices by a combined ATP/DNA quantification assay.

**Results:**

We obtained a mean of 49 tissue slices from 22 breast cancer specimens allowing a wide range of experiments in each individual tumor. In our culture system, cells remained viable and proliferated for at least 4 days within their tissue environment. Viability of tissue slices decreased significantly in the presence of taxol in a dose-dependent manner. A three-color fluorescence viability assay enabled a rapid and authentic estimation of cell viability in the different tumor compartments within non-fixed tissue slices.

**Conclusion:**

We describe a tissue culture method combined with a novel read out system for both tissue cultivation and rapid assessment of drug efficacy together with the simultaneous identification of different cell types within non-fixed breast cancer tissues. This method has potential significance for studying tumor responses to anticancer drugs in the complex environment of a primary cancer tissue.

## Background

It is becoming increasingly evident that the development of cancer and response to anticancer drug therapy not only depend on discrete genetic alterations in the malignant clone but also on specific interactions between tumor cells and surrounding tissue components. The mammary gland is composed of different cell types and extracellular matrix proteins [1]. In the normal gland, luminal epithelial cells in the ducts are encased by myoepithelial cells which are in contact with a basement membrane. This intact basement membrane separates epithelial cells from a surrounding highly compartmentalized stroma which accounts for more than 80% of the normal breast volume [2]. Conversely, in invasive carcinoma, fully differentiated myoepithelial cells and intact basement membranes are often lost and tumor cells are in direct contact with a highly activated collagenous tumor-stroma [3,4]. Our understanding of interactions between epithelium and stroma within the cancerous mammary gland and their role for drug responsiveness is still rudimentary. Obviously, this is because most established *in vitro *models fail to reflect the complex tissue architecture of an individual tumor.

The majority of preclinical breast cancer research is based on established cell lines [5]. However, these cell lines frequently have undergone multiple changes influencing their biological behavior and therefore no longer reflect the primary tumor of origin. Freshly isolated primary epithelial cells, in contrast, may be more closely related to the malignant epithelial cells of the tumor [5]. Also, it is difficult to adapt the cells of many tumors to *in vitro *conditions when establishing a primary epithelial culture. In addition, it is most likely that separated tumor cells will behave differently *in vitro*, as both cell-cell and cell-matrix interactions are highly different compared to the *in vivo *situation. Therefore, to investigate tumor cell behavior *ex vivo *it is necessary to maintain or reconstitute an environment closely resembling the tumor tissue. To simulate such conditions either three-dimensional tissue cultures using several biomatrices or co-culture experiments with tumor fibroblasts have been performed [6,7]. These studies have provided important information concerning both the impact of communication between tumor cells and fibroblasts and the interaction between extracellular matrix, integrins, and various intracellular signal cascades in epithelial cells [7-10]. However, these systems can not mimic the complex tissue architecture and the high degree of variability seen in individual tumors. One possibility to maintain the tissue architecture *ex vivo *is the direct cultivation of fresh and intact tumor material. First experiments in this direction were performed in 1967 by Matoska and Stricker using tumor cubes of approximately 1 mm^3 ^[11]. A problem that arose in this approach was restricted diffusion of oxygen and nutrients leading to cell death in the center of the tissue cubes. This was overcome by the introduction of a tissue microtome enabling the preparation of thin tissue slices [12-15]. We have improved this tissue culture system to study the activity of anticancer drugs such as taxol in different tissue compartments. The use of a confocal laser scan microscopy based technique enabled us to identify cell type and test cell viability in tissue slices maintained for at least 4 days in culture.

## Methods

### Tissue slice preparation and culture

**Figure 1:**
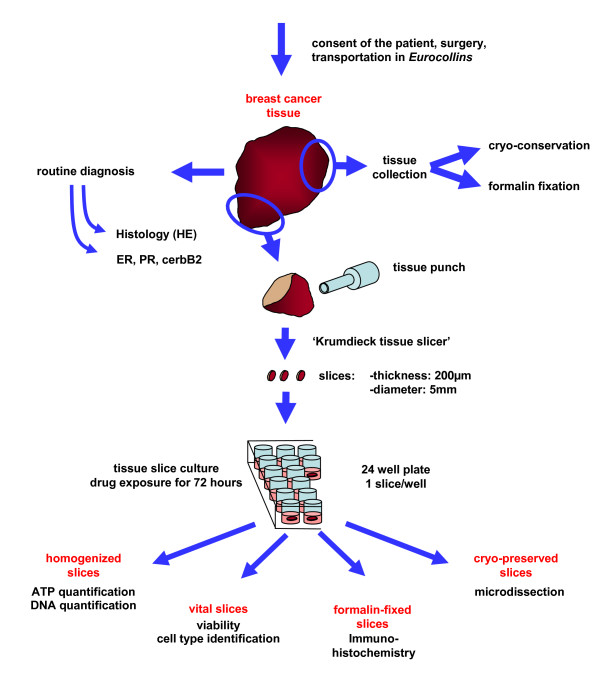
Procedure of slice preparation and tissue cultivation.

Fresh sterile tissue of primary breast tumors larger than 3 cm were obtained as surgical waste from patients newly diagnosed for breast cancer of invasive ductal type (n = 24) and mucinous type (n = 1) at the Robert Bosch Hospital immediately after surgical resection and maintained in organ transportation medium (Eurocollins, Fresenius medical care, Bad Homburg, Germany) on ice until use. This investigation was approved by the local ethics committee and informed consent was obtained from the patients. Patients treated with neoadjuvant chemotherapy were excluded. Tissue slice preparation is illustrated in figure 1. Tissue cores (5 mm in diameter) were prepared under a sterile hood using a hand held coring tool. From the cylinders, tissue slices with a thickness of 200μm were prepared in cold PBS using a precision cutting tissue slicer (Krumdieck, Alabama Research and Development Corp., Munsford, USA). To avoid contamination, all parts of the tissue slicer which have contact to the tissue or buffer were cleaned with 70% 2-propanol and steam sterilized before use. Slices were then individually submerged in 1 ml Mammary Epithelial Growth Medium supplemented with bovine pituary extract, 10 ng/ml rhEGF, 5μg/ml bovine Insulin, 500 ng/ml hydrocortisone, 100μg/ml gentamicin, and 0.05μg/ml amphotericin B (PromoCell, Heidelberg, Germany). Incubation was performed in 24-well plates at 37°C in a constant atmosphere of 5% CO_2 _on a shaking platform with 150 rpm (Titramax 100, Heidolph, Schwabach, Germany). Medium was changed every 24 hours. Treatment with taxol started after 24 hours and was performed for an additional 72 hours. Taxol concentrations were selected according to test drug concentrations from a commercially available standard tumor chemosensitivity assay (TCA-100; DCS Innovative Diagnostic systems, Hamburg, Germany). To investigate cell proliferation, slices were incubated with 100μM BrdU and 1μM FdU for 4 h. Slices were then either transferred in a 2 mM EDTA solution (pH = 10.9) in ethanol (70% v/v) and immediately frozen in liquid nitrogen, fixed in formalin, cryo-conserved, or stained with fluorescent dyes for identification of cell type and viability.

### Simultaneous identification of living and dead cells within tissue slices

To identify living and dead cells within the non-fixed tissue slices, we established a three-color and two-color fluorescent viability assay. Living cells were detected using tetramethylrhodamine methyl ester (TMRM; Molecular Probes, Invitrogen, Karlsruhe, Germany). SYTO^®^63 (Molecular Probes, Invitrogen) is a low-affinity nucleic acid stain that passively diffuses through the membranes of living and dead cells. Picogreen (Molecular Probes, Invitrogen) or propidium iodide (PI; Sigma-Aldrich, Deisenhofen, Germany) on the other hand, are DNA-selective dyes that are membrane impermeant but that easily pass through the compromised plasma membranes of dead cells. For three-color fluorescent staining, cells or tissues were incubated simultaneously with 0.5μM TMRM, 1.25μM SYTO^®^63, and a concentrated solution of Picogreen in DMSO (Molecular Probes, Invitrogen) in a 1:1500 dilution in the culture medium at 37°C for 45 min and analyzed immediately without further washing steps using confocal microscopy. The two-color fluorescent stain assay was performed using 1.25μM SYTO^®^63 and either Picogreen (1:1500) or 0.2μg/ml PI in a PBS/1% BSA solution for 30 min and examined immediately or following additional antibody staining using confocal microscopy.

### Identification of different tumor compartments within non-fixed tissue slices

Epithelial cells and endothelial cells were distinguishable using fluorescent-labeled antibodies recognizing specific surface markers. Slices were transferred in 1 ml PBS containing 1% BSA and 1.25μM SYTO^®^63. After an incubation period of 30 min at 37°C, the volume was reduced to 100μl and conjugated antibody or *Ulex europaeus *Agglutinin I (UEA-1) was added. Epithelial cells were identified with a FITC-conjugated anti-HEA-125 antibody (1:20; Biomeda, Foster City, CA). To visualize the vascular network, fresh tissue slices were directly labeled using FITC-conjugated UEA-1 (1:50; Alexis, Grunberg, Germany) or phycoerythrin (PE)-conjugated CD34 antibody (1:20; BD Biosciences Pharmingen, Heidelberg, Germany). Slices were incubated with antibody or UEA-1 for 20 min at 4°C. The staining solution was removed and slices were washed with cold PBS/BSA containing PI (0.2μg/ml) for 10 min. Slices were then transferred on microscope slides and cells visualized using a confocal microscope with a 40× objective (Leica Lasertechnik, Wetzlar, Germany).

### Confocal microscopy and triple-fluorescence analysis

Confocal laser scanning microscopy was performed using a Leica LCS (Leica Lasertechnik) instrument based on a Leica DM IRBE microscope interfaced with argon and helium/neon lasers emitting at 488 nm, 543 nm and 633 nm. To separate the detection channels we used a spectrophotometer. The different colors were detected sequentially at 500–520 nm for Picogreen and FITC, 560–590 nm for TMRM, PE, and PI, and 650–700 nm for SYTO^®^63.

### Quantification of triple-fluorescence viability assay

Confocal images were counted by two independent observers. The numbers of TMRM^+^, SYTO^®^63^+^, and Picogreen^+ ^cells were evaluated by direct counting of at least 50 cells from at least two different areas of the tissues in 400× magnification images. Counts were expressed as mean number (averaged between the observers) of TMRM^+^, SYTO^®^63^+^, and Picogreen^+ ^cells per image. The ratio of vital cells (mean of TMRM^+ ^and SYTO^®^63^+ ^cells/mean of total cells) and dead cells (mean of Picogreen^+ ^cells/mean of total cells) was evaluated for each individual tumor.

### Immunohistochemical staining

Tissue slices were fixed in 10% phosphate-buffered formalin. For histopathological examination paraffin sections (3μm) were stained with hematoxylin and eosin. Immunohistochemical staining for CD34 (M7165; DakoCytomation, Hamburg, Germany; 1:50), BrdU (anti-BrdU clone BU-33, B2531, Sigma-Aldrich; 1:100), HEA (M0804, DakoCytomation; 1:100) or Caspase 3 cleavage product (#9661, Cell Signal Technology, Beverly, MA; 1:50) was performed using the Dako Envision Kit on a DakoCytomation Autostainer (both DakoCytomation) according to the manufacturer's manual. Epitope retrieval was achieved as follows: prior to staining for CD34, HEA, and Caspase 3 cleavage product by treatment in a steam heater for 15 min, and by incubation in 2 M HCl/0.1 M borax at 37°C for 30 min followed by incubation with pronase at 37°C for 30 min before staining for BrdU. Counterstaining was performed with hematoxylin.

### ATP quantification

Tissue slices were transferred into a 2 mM EDTA solution (pH = 10.9) in ethanol (70% v/v) and immediately frozen in liquid nitrogen. The slices were homogenized using *lysing matrix D *and a *FastPrep *instrument (Qbiogene, Heidelberg, Germany). 50μl of slice homogenate were transferred into 450μl phosphate buffer (0.1 M, pH = 7.5). The content of ATP was determined in this solution using ATP Bioluminescence Assay Kit (DSC, Hamburg, Germany). To correct for cell numbers within individual slices, DNA content was measured in parallel using the Picogreen DNA quantification system (Molecular Probes).

### Statistics

Calculation of means, standard deviation (SD), and standard error of the mean (SEM) was done in GraphPad Prism (V 3.0, GraphPad Prism Software Incorp., San Diego, CA, USA). Different groups were compared by Kruskal-Wallis test.

## Results

### Preparation of tissue slices

Circular punches of fresh tumor material were cut into 200μm slices in ice cold phosphate buffered saline (PBS) using a Krumdieck microtome. These slices were individually submerged in 1 ml Mammary Epithelial Growth Medium. Details of this procedure are illustrated in figure 1. Viable tissue slices were obtained from 22 of 25 breast tumor samples. In one case the cells were completely dead because of a prolonged transportation without transportation medium (Eurocollins), one was contaminated with bacteria, and a mucinous carcinoma was too soft to cut. Depending on the size of the individual tumor sample a mean of 49 viable tissue slices (range: 24 – 130) were obtained.

### Identification of living and dead cells within non-fixed tissues

**Figure 2:**
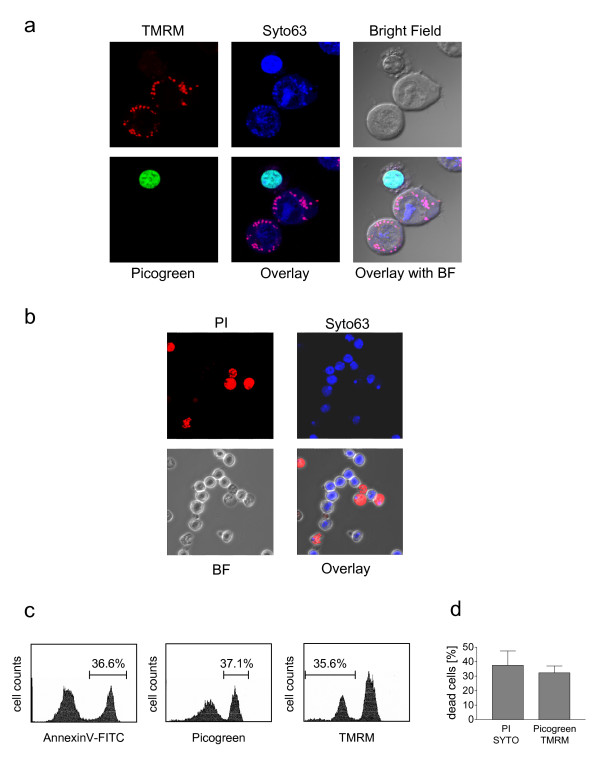
**Principle of three and two-color fluorescence viability assay**: Bcr-Abl positive BaF3 cells were cultivated in the presence of 1μM Imatinibfor 8 hours. (**a**) Following Imatinib treatment cells were incubated with TMRM (given color: red), SYTO^®^63 (given color: blue), and Picogreen (given color: green) and visualized using a confocal microscope with a 63X objective. (**b**) Following Imatinib treatment cells were incubated in a PBS-BSA solution containing PI (given color: red) and SYTO^®^63 (given color: blue) and visualized by confocal microscopy. (**c**) Comparison of cell death indices determined by Annexin V-FITC staining or TMRM/Picogreen staining in Imatinib treated cells by means of FACS analysis. (**d**) Comparison of cell death indices determined by PI/SYTO63 staining or TMRM/Picogreen staining. Cells were visualized by confocal microscopy with a 40× objective. 10 different areas with each at least 20 cells were counted. Values are means of the ratio PI^+ ^cells or Picogreen^+ ^cells to total cells ± SD from 10 different areas.

To identify living and dead cells within non-fixed tissue slices, we established a two- and three-color fluorescent viability assay composed of SYTO^®^63, and Picogreen or PI for both assays and in addition TMRM for the three-color assay. Living cells were detected with TMRM, a probe that accumulates within polarized mitochondria, wherefrom it is released completely upon depolarization. Picogreen and PI are DNA-selective dyes that are membrane nonpermeant but that easily pass through the compromised plasma membranes of dead cells. Thus, these dyes selectively detect dead cells. SYTO^®^63 on the other hand is a lower-affinity nucleic acid stain that passively diffuses through the membranes of living and dead cells. Cells or tissues can be incubated simultaneously with these dyes within the appropriate medium at 37°C for 45 min and analyzed immediately using confocal microscopy. We evaluated these methods using a Bcr-Abl positive hematopoietic cell line treated with Imatinib (STI571) for a short time period. Imatinib selectively kills Bcr-Abl positive cells [16]. As shown in figure 2a TMRM accumulates in mitochondria of living cells and cannot be detected in dead cells. Dead cells are characterized by a bright green fluorescence due to Picogreen/DNA binding in the three-color fluorescent assay and a red fluorescence due to PI/DNA binding in the two-color fluorescent assay (Fig. 2a and 2b). All cells are stained by SYTO^®^63 (Fig. 2a and 2b). The comparison of the three-color fluorescent assay with a widely accepted method for quantification of cell death (Annexin V-FITC staining) by means of FACS analyses revealed similar results (Fig. 2c). In addition, visually counted evaluation of the three- and two-color based assay using a confocal microscope gave equal results (Fig. 2d). Therefore, these assays allow a precise, simple, and rapid estimation of viable and dead cells within non-fixed tissue slices by confocal microscopy.

**Figure 3:**
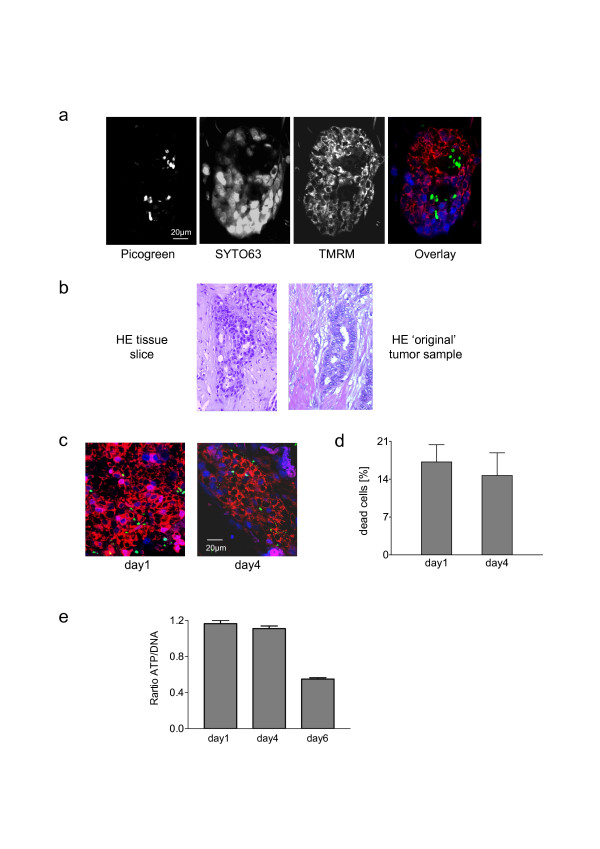
**Viability of cultivated tissue slices**: Tumor slices obtained from a breast carcinoma were individually cultivated in 1 ml medium for 24 hours. Slices were then analyzed for cell viability or cultivated for additional 72 hours or 144 hours as indicated. (**a**) Representative example of a tissue slice stained with Picogreen, SYTO^®^63, and TMRM. (**b**) Hematoxylin and eosin (HE) staining of a section from a paraffin embedded tissue slice after a culture period of 96 hours in comparison to that observed in a section obtained from the same tumor directly after surgery ('original' tumor sample). (**c**) Cell viability after an *ex vivo *cultivation period of 24 and 96 hours determined by TMRM, SYTO^®^63, and Picogreen labeling. (**d**) Quantification of cell death (number of visually counted Picogreen^+ ^cells in relation to the number of total cells in 3 different areas) in non fixed tumor slices from 6 patients. Values reflect means ± SEM (n = 6). (**e**) Ratio of luminescence based quantification of ATP to DNA content of homogenates from slices cultivated for 24, 96, or 144 hours. (Data of a representative experiment shown).

In tissue samples from breast tumor specimens SYTO^®^63 and TMRM labeled cells reflecting living cells were highly concentrated throughout the slice. Figure 3a shows a representative example of a tumor area stained with Picogreen, SYTO^®^63 and TMRM. In analogy to the results obtained with single cells (Fig. 2a), Picogreen and TMRM staining was found to be mutually exclusive in tissue slices (Fig. 3a). Histological examination of paraffin embedded tissue slices cultivated for 4 days revealed no obvious differences in the morphology when compared to the paraffin embedded 'original' tumor tissue from routine diagnosis (Fig. 3b). TMRM staining depends on the proper functioning of the mitochondria. The mitochondrial metabolism may be influenced by growth factors [17] and by low temperature. Following surgery the tissues are kept in ice cold transportation medium (Eurocollins) lacking growth factors until cultivation. The time frame between surgery, preparation by the pathologist, slicing, and cultivation was at least 3 hours. We therefore avoided starting our experiments immediately after slice preparation. Within 24 hours of cultivation, TMRM labeling identified a high percentage of vital cells without significant changes during the following 72 hours (representative images in Fig. 3c). This was confirmed by quantification of Picogreen^+ ^cells in relation to total cells in 6 tumor samples. The median ratios of dead cells at days 1 and 4 were 17.6% and 14.6%, respectively (Fig. 3d). In addition, the ATP/DNA ratio from homogenized slices did not change significantly during the time frame between day 1 and day 4 of culture. A prolonged cultivation, however, turned out to be ineffectual: In the majority of the cases investigated there was a gradual decline of viability starting at day 5 (representative result in Fig. 3e).

### *Ex vivo *treatment of tissue slices with taxol

**Figure 4:**
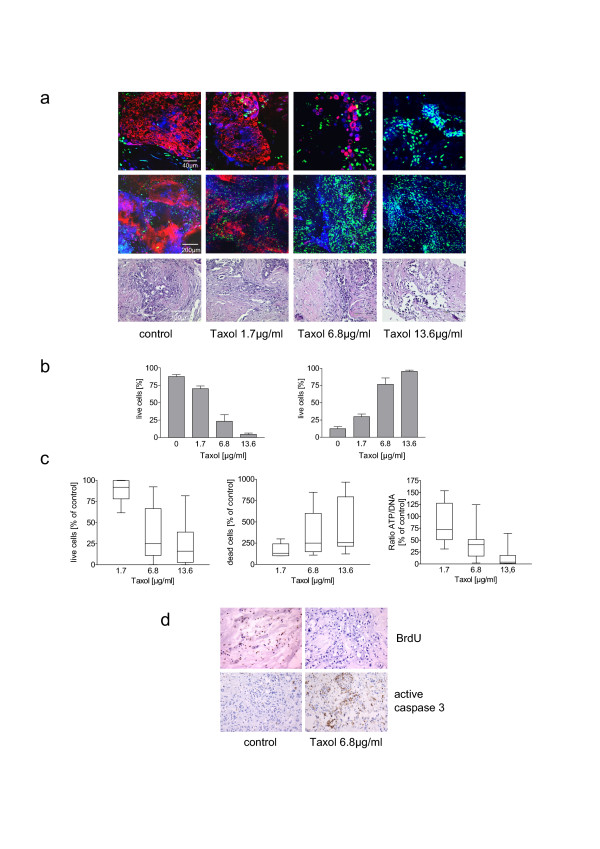
**Influence of Taxol treatment on cell viability**: (**a**)Three-color fluorescence viability assay (top and middle) and hematoxylin/eosin staining (bottom) of slices treated with different taxol concentrations for 72 hours (images of a representative experiment). (**b**) The numbers of TMRM^+^, SYTO^®^63^+^, and Picogreen^+ ^cells in tumor slices from (a) were evaluated by counting of at least 50 cells from three different areas of different images. Viable cells were assessed as numbers of TMRM^+ ^and SYTO^®^63^+ ^cells in relation to total cells (left panel). Dead cells were evaluated as numbers of Picogreen^+ ^cells in relation to total cells (right panel). Values reflect means ± SEM. (**c**) Dose dependent effect of taxol treatment on cell viability determined by quantification of TMRM^+^, SYTO^®^63^+^, and Picogreen^+ ^cells in non fixed tumor slices (left and middle panel) and by evaluation of ATP/DNA ratio in slice homogenates (right panel). Ratios of controls were set to 100%, and the relative ratios of the taxol treated tissues were calculated. Data represent range, median and 25 to 75% percentile of a set of 10 experiments. Kruskal-Wallis test: p < 0.0001 for all three. (**d**) Effect of taxol treatment on proliferation and apoptosis. Formalin-fixed and paraffin-embedded slices were stained with anti-BrdU or anti-caspase 3 antibodies by conventional immunohistochemistry and counterstained with hematoxylin.

To study the activity of an anticancer drug in this tissue culture model we treated 10 tumor samples with different concentrations of taxol for 72 hours. Figure 4a shows a representative result of one sample evaluated both by three-color viability assay and histological examination. TMRM accumulation was significantly reduced (images shown in Fig. 4a and quantification in Fig. 4b, left panel) while nuclear staining of dead cells with Picogreen increased following treatment of tissue slices with taxol in a dose-dependent manner (Fig. 4a and Fig. 4b, right panel). The low magnification corresponds to approximately 10% of the total slice area and is representative for the complete tissue slice demonstrating that almost all cells die in the presence of higher taxol concentrations in this tumor sample (Fig. 4a). Histological examination revealed an increasing number of disintegrated cells in the presence of high taxol concentrations (Fig. 4a). The overall response to taxol in the 10 tumor samples investigated is shown in figure 4c. The proportion of viable cells (ratio TMRM^+ ^and SYTO^®^63^+ ^cells to total cells; Fig. 4c, left panel) decreased whereas dead cells (ratio Picogreen+ cells to total cells; Fig. 4c, middle panel) increased significantly in a dose-dependent manner. This result was mirrored by quantification of the ATP/DNA ratio in slice homogenates (Fig. 4c right panel). The response to taxol determined for each individual tumor turned out to be heterogeneous. One tumor sample was almost completely resistant to taxol whereas 6 tumors turned out to be highly sensitive to higher taxol concentrations: incubation of slices from these tumors in the presence of 6.8μg/ml or 13.6μg/ml taxol for 4 days induced cell death in almost all cells as determined by an almost complete staining with Picogreen and by ATP breakdown. An intermediate response to taxol was observed in 3 tumors.

The activity of taxol was also determined in sections from paraffin embedded slices by immunohistochemical detection of active caspase 3 and BrdU incorporation. Treatment of slices with taxol led to a decrease of BrdU positive cells and an increase of active caspase 3 (Fig. 4d).

### Identification of tumor compartments

**Figure 5:**
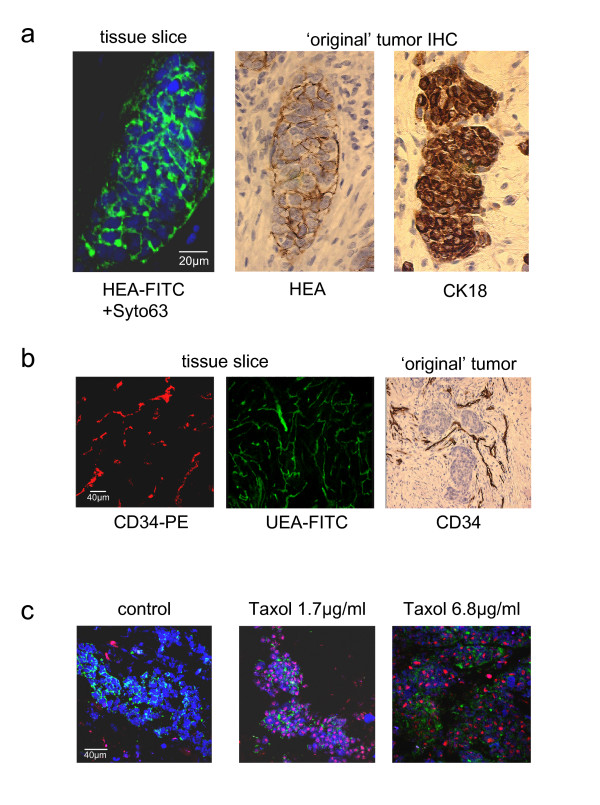
**Identification of different cell types within tumor slices**: (**a**) epithelial cells were identified using an anti-HEA-125 antibody recognizing an epithelial specific adhesion molecule (Ep-CAM). The morphology of epithelial cell clusters in slices cultivated for 96 hours (left panel) was compared to that observed in sections from paraffin embedded material from the same tumor prepared immediately after surgery labeled either with anti-HEA-125 (middle panel; counterstained with hematoxylin) or anti-cytokeratin 18 (right panel; counterstained with hematoxylin). (**b**) To visualize the vascular network tissue slices cultured for 96 hours were directly labeled using a PE-conjugated CD34 antibody (left panel) or a FITC-conjugated *Ulex europaeus *Agglutinin I (UEA-1; middle panel). The morphology of this network is comparable to that observed in paraffin embedded material obtained directly after surgery and stained with CD34 antibody (right panel, counterstained with hematoxylin). (**c**) Simultaneous staining of epithelial cells and determination of cell viability in slices treated with or without taxol for 72 hours. Epithelial cells were identified using a FITC-conjugated anti-HEA-125 antibody (green). Cell viability was determined using the two DNA selective dyes PI (red) and SYTO^®^63 (blue).

As shown in figure 5a (left panel), epithelial cells can be identified using a fluorescent-labeled antibody recognizing Ep-CAM (anti-HEA-125 antibody) in viable tissues. The morphology of the epithelial compartment was identical to that observed in the same tumor examined immediately after surgery by conventional immunohistochemistry using HEA antibody (Fig. 5a, middle panel) or cytokeratin 18 (CK18) antibody as epithelial markers (Fig. 5a, right panel).

The vascular network was visualized by staining slices with PE-labeled anti-CD34 antibody or FITC-conjugated UEA-1. As shown in figure 5b the morphology of this network was comparable to that observed in paraffin embedded material stained with endothelial-specific antibodies, such as CD34 [18].

To simultaneously identify epithelial cells and investigate cell viability following taxol treatment the two-color fluorescent viability assay consisting of SYTO^®^63 and PI was combined with the detection of Ep-CAM. Almost all epithelial cells (green) were viable in the control (negative for PI) whereas epithelial cells (green) were positive for nuclear PI staining (red) in taxol treated slices (Fig. 5c). The tumor shown in this figure was highly sensitive to taxol as many dead epithelial cells were detected even at the lowest concentration used.

## Discussion

Although most of the research into cancer drug sensitivity *ex vivo *was initially based upon disaggregated tumors and single cell culture experiments [19,20], it has now become clear that the tumor environment has a wide influence on the resistance of cancer cells to therapy [21]. Cell-cell and cell-matrix interactions responsible for this impact have been studied in 2D and 3D *in vitro *culture models [22-24], in spheroid models [25,26] and in co-culture experiments using immortalized tumor cell lines and primary fibroblasts [27]. However, these interactions are likely to be extremely complex and specific for each individual tumor *in vivo *[21]. It is therefore of great interest to advance tissue culture models for studying the activity of anticancer drugs and small molecule inhibitors in an intact tumor environment of individual tumors – particularly as there are different targets within the tumor tissue: the epithelial tumor cells themselves and the surrounding non-tumorgenic cell types. We have combined a tissue culture method described by Krumdieck et al. [14] and Hood & Parham [15] with a novel read out system for a rapid assessment of drug efficacy together with the simultaneous identification of different cell types within the fresh tissue material. With this culture technique it was possible to cultivate freshly excised tumor material from 22 of 25 patients in the presence or absence of drugs *ex vivo *for at least 4 days without significant loss of cell viability. Therefore, this technique may provide a valid tool to investigate drug resistance and effectiveness of anticancer drugs in a large number of tumor samples. The accurately defined thickness of the tissue slices (200μm) allows a smooth diffusion of nutrients, drugs, and antibodies. Fluorescent labeled taxol and antibodies were found to be distributed throughout the slice (Additional File 1). Following treatment, the tissues can be analyzed on different levels (Fig. 1). Non fixed slices stained with TMRM, SYTO^®^63, Picogreen/PI, and/or fluorescent-conjugated antibodies for estimation of drug sensitivity and identification of cell type can be further utilized for conventional immunohistochemistry. After formalin-fixation it is possible to take multiple 3μm sections from the paraffin embedded slices at later date. It is also feasible to prepare cryo-sections from frozen tissue slices for laser capture microdissection allowing the separation of the different cellular tumor compartments to analyze them separately via genomic or proteomic approaches. Furthermore, homogenized slices can be used for ATP and DNA quantification to assess the overall viability of the tumor tissue. In addition, the culture supernatant can be analyzed for metabolites and peptides or proteins released as a consequence of cell death such as CK18 [28].

Furthermore, this *in vitro *culture system provides a tool for studying the differential responses of specific tumor compartments to anticancer drugs and may therefore e.g. allow evaluating whether manipulation of the stromal compartment alters drug response of tumor cells. This is of utmost importance for the development of novel combinatorial strategies involving novel pharmacological compounds such as signal transduction inhibitors and interfering RNAs (siRNAs), particularly as these substances specifically target either the tumor or the stromal compartment. Together with well established models such as 3D culture systems and animal tumor xenografts, this tissue slice model will be helpful to enhance the understanding of anti-tumor drug activity.

## Conclusion

This method has potential significance for studying tumor responses in the complex environment of a primary cancer tissue enabling a molecular profiling of all tumor compartments using laser microdissection techniques. Subsequently, it can be used to analyze the molecular response of each tissue component to both cytotoxic drugs and signal transduction inhibitors via genomic or proteomic approaches. Therefore, this method opens the window for extensive molecular studies on the biological effects of conventional and innovative treatment strategies.

## Abbreviations

epithelial cell adhesion molecule (Ep-CAM); human epithelial antigen (HEA); propidium iodide (PI); phycoerythrin (PE); tetramethylrhodamine methyl ester (TMRM); *Ulex europaeus *Agglutinin I (UEA-1)

## Competing interests

The author(s) declare that they have no competing interests.

## Authors' contributions

HvdK, TEM, and MS were responsible for the development of the culture method and the three-color fluorescent assay. HvdK, TEM, MS, MM, and SG performed the experiments. MS, MM, and SG were responsible for immunostaining. HvdK, TEM, MS, and PF were accountable for data evaluation. HvdK, TEM, and WEA were responsible for the design of the experiments and for writing the manuscript. WS, AG, and PF were responsible for surgery, patient information, and collection of tissue material. All authors read and approved the final manuscript.

## Pre-publication history

The pre-publication history for this paper can be accessed here:



## Supplementary Material

Additional File 1 **Diffusion of taxol and antibodies in tissue slices: **confocal stack consisting of a series of single digital images top down of a tissue slice stained with oregon-green taxol (a) or FITC-conjugated anti-HEA-125 antibody (b). The series were taken at 1 image/3 μm. Click here for file
