# Supplementary figures and images for: Short term culture of breast cancer tissues to study the activity of the anticancer drug taxol in an intact tumor environment
Source: BMC Cancer. 2006 Apr 7;6:86. doi: 10.1186/1471-2407-6-86 (PMC1456977; doi:10.1186/1471-2407-6-86)

a

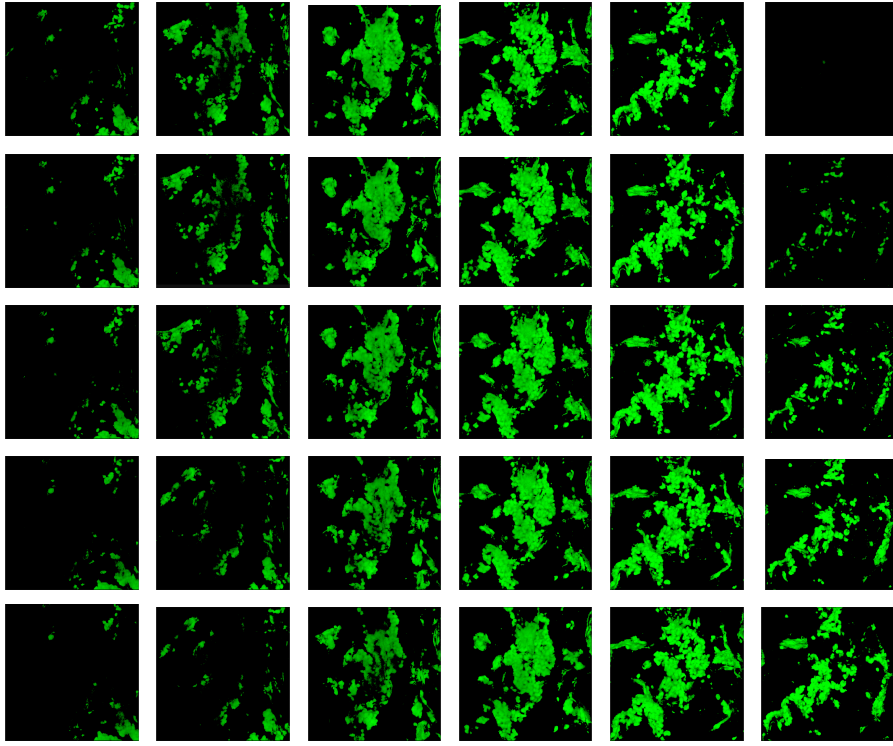

b

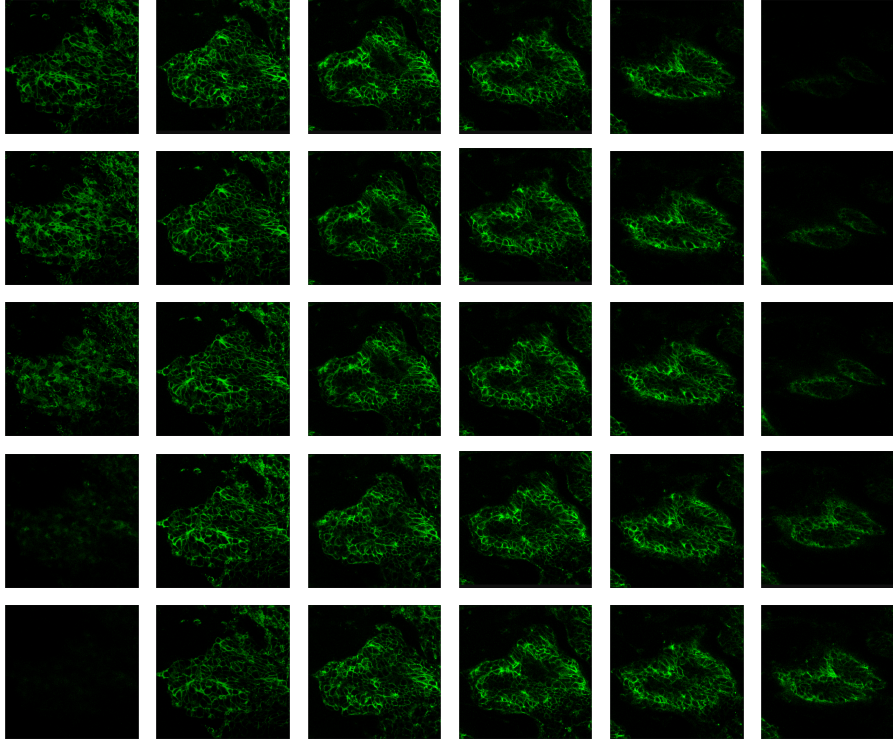

Supplement: Additional File 1 — Diffusion of taxol and antibodies in tissue slices: confocal stack consisting of a series of single digital images top down of a tissue slice stained with oregon-green taxol (a) or FITC-conjugated anti-HEA-125 antibody (b). The series were taken at 1 image/3 μm. [file 1471-2407-6-86-S1.pdf]
